# Genomic and environmental controls on *Castellaniella* biogeography in an anthropogenically disturbed subsurface

**DOI:** 10.1186/s40793-024-00570-9

**Published:** 2024-04-26

**Authors:** Jennifer L. Goff, Elizabeth G. Szink, Konnor L. Durrence, Lauren M. Lui, Torben N. Nielsen, Jennifer V. Kuehl, Kristopher A. Hunt, John-Marc Chandonia, Jiawen Huang, Michael P. Thorgersen, Farris L. Poole, David A. Stahl, Romy Chakraborty, Adam M. Deutschbauer, Adam P. Arkin, Michael W. W. Adams

**Affiliations:** 1 Department of Biochemistry and Molecular Biology, University of Georgia, Athens, GA 30602 USA; 2 Present Address: State University of New York College of Environmental Science and Forestry, Syracuse, NY USA; 3 Environmental Genomics and Systems Biology, Lawrence Berkeley National Laboratory, Berkeley, CA USA; 4 Department of Civil and Environmental Engineering, University of Washington, Seattle, WA USA; 5 Earth and Environmental Science Area, Lawrence Berkeley National Laboratory, Berkeley, CA USA; 6 Department of Bioengineering, University of California-Berkeley, Berkeley, CA USA

**Keywords:** Contamination, Pangenome, Mobile genetic elements, Heavy metals, Nitrate, Acid tolerance

## Abstract

*Castellaniella* species have been isolated from a variety of mixed-waste environments including the nitrate and multiple metal-contaminated subsurface at the Oak Ridge Reservation (ORR). Previous studies examining microbial community composition and nitrate removal at ORR during biostimulation efforts reported increased abundances of members of the *Castellaniella* genus concurrent with increased denitrification rates. Thus, we asked how genomic and abiotic factors control the *Castellaniella* biogeography at the site to understand how these factors may influence nitrate transformation in an anthropogenically impacted setting. We report the isolation and characterization of several *Castellaniella* strains from the ORR subsurface. Five of these isolates match at 100% identity (at the 16S rRNA gene V4 region) to two *Castellaniella* amplicon sequence variants (ASVs), ASV1 and ASV2, that have persisted in the ORR subsurface for at least 2 decades. However, ASV2 has consistently higher relative abundance in samples taken from the site and was also the dominant blooming denitrifier population during a prior biostimulation effort. We found that the ASV2 representative strain has greater resistance to mixed metal stress than the ASV1 representative strains. We attribute this resistance, in part, to the large number of unique heavy metal resistance genes identified on a genomic island in the ASV2 representative genome. Additionally, we suggest that the relatively lower fitness of ASV1 may be connected to the loss of the nitrous oxide reductase (*nos*) operon (and associated nitrous oxide reductase activity) due to the insertion at this genomic locus of a mobile genetic element carrying copper resistance genes. This study demonstrates the value of integrating genomic, environmental, and phenotypic data to characterize the biogeography of key microorganisms in contaminated sites.

**Supplementary Information:**

The online version contains supplementary material available at 10.1186/s40793-024-00570-9.

## Introduction

The nitrogen cycle has been profoundly impacted by agricultural and industrial activity, with excess nitrogen deposition contributing to the eutrophication of rivers, increased greenhouse gas emissions, and pollution of subsurface water sources [28, 64]. The Oak Ridge Reservation (ORR) in Oak Ridge, Tennessee is an extreme example of nitrate contamination, where concentrations in the subsurface reach up to 190 mM as a consequence of nuclear materials processing throughout the mid-twentieth century [102]. The ORR Y-12 plant generated millions of liters of waste containing nitric acid and metals such as copper (Cu), cadmium (Cd), cobalt (Co), nickel (Ni), and uranium (U). Much of this liquid waste was deposited in an unlined surface disposal area (designated the S-3 ponds) between 1951 and 1983, resulting in extensive contamination of the subsurface [19]. Although attempts to remediate the site have been pursued, the subsurface environment surrounding the former S-3 ponds remains highly acidic and contains elevated levels of various toxic metals and nitrate [19, 97–99, 102]. Despite the extreme conditions of the ORR subsurface, persistent microbial communities have been identified via metagenome surveillance and further characterized in culture-dependent studies [40, 44, 98, 99]. Community members include large proportions of nitrate-reducing and denitrifying species [40, 44, 97]. Thus, developing an understanding of the abiotic and genomic controls on microbially driven nitrate reduction and nitrogen cycling at this site is important for predicting the fate of the contaminating nitrate.

A study examining the effects of electron donor injection on subsurface community structure and nitrate reduction activity at ORR found that biostimulation diminished overall microbial diversity, leading to an increase in Betaproteobacteria. Many of these Betaproteobacteria are capable of nitrate reduction, specifically members of the *Castellaniella* genus, suggesting that this genus may play a key role in nitrate remediation [98, 99]. *Castellaniella* strains have also been found in a variety of built and natural environments including forest soils [33], microbial fuel cells [4], and sludges from wastewater treatment plants [30, 70]. These *Castellaniella* isolates are metabolically versatile and remarkably well-adapted for life in anthropogenically disturbed environments. For example, *Castellaniella* isolates have been shown to degrade cyclic monoterpenes under anoxic conditions [84], as well as to utilize taurine [27] and phenol [88] as their sole electron donors under anoxic, nitrate-reducing conditions. Thus, this genus represents a relevant model for studying mechanisms of adaptive evolution and the influence of environmental stressors on diversification events.

In this study, we present an analysis of *Castellaniella* diversity of the ORR subsurface within the broader context of other members of the *Castellaniella* genus. For this analysis, we asked the following questions: (1) What is the geographic distribution of *Castellaniella* diversity in the ORR subsurface? (2) Does variation in phenotypes observed during laboratory simulations of relevant environmental stress explain the distribution and the differential abundance of distinct *Castellaniella* clades within the ORR subsurface? To address these questions, we integrated environmental field, genomic, and phenotypic data to examine site-relevant traits of ORR *Castellaniella* isolates.

## Materials and methods

### Bacterial strains

Experimental work and genome sequencing were performed with six *Castellaniella* strains isolated from groundwater samples collected at the Oak Ridge Reservation (ORR) in Oak Ridge, Tennessee, USA. *Castellaniella* sp. str. MT123 was previously isolated from groundwater samples collected from the contaminated well FW104 (lon. 35.97736048°, lat. − 84.27356212°), adjacent to the former S-3 waste ponds at the Field Research Center in Oak Ridge, TN [102]. *Castellaniella* strains FW104-7C03, FW104-12G02, FW104-16D08, and FW104-7G2B were also isolated from this FW104 groundwater, and *Castellaniella* strain GW247-6E4 was isolated from groundwater taken from well GW247 (lon. 35.97729963°, lat. − 84.27272624°) at ORR. Both wells are impacted by the contamination plume in the ORR subsurface [96]. FW104-7C03, GW247-6E4, and FW104-7G2B were isolated on TSA plates under aerobic growth conditions at 30 °C. FW104-12G02 was isolated on Eugon agar plates under aerobic growth conditions at 30 °C. FW104-16D08 was isolated on LB agar plates under aerobic growth conditions at 30 °C.

### Aerobic phenotyping conditions

*Castellaniella* cultures were grown overnight by inoculating LB broth with five individual colonies grown on R2A plates. The overnight cultures were grown at 30 °C while shaken at 200 rpm. For aerobic growth experiments, 5 µL subsamples of the overnight cultures were inoculated into 200 μL of *Castellaniella* Experimental Medium  in individual wells of 96-well plates. CEM contains, per liter, 0.6 g NaH_2_PO_4_, 20 mL of 1 M sodium lactate (20 mM final concentration), 40 mL of a 25X salts solution, and 1 mL of a 1000X DL Vitamins stock and 10 mL of a 100X DL minerals stock as described previously in Widdel and Bak (1992). The 25X salts solution contains, per liter, 250 mg NaCl, 367 mg CaCl_2_ ∙ 2H_2_O, 12.32 g MgSO_4_ ∙ 7H_2_O, 2.5 g KCl, and 5 g NH_4_Cl. The pH of the medium was adjusted based on the desired conditions with HCl or NaOH. Different buffers were used based on the desired culture pH. For cultures that were grown at pH < 7, we added 1 mL of 2 M sodium acetate buffer per liter. Control experiments using the acetate buffer with lactate excluded did not yield significant growth. For cultures that were grown at pH ≥ 7, a bicarbonate buffer (2.5 g of NaHCO_3_ per liter) was utilized. The growth medium was sterilized by vacuum filtration. Aerobic growth experiments included the growth of *Castellaniella* isolates at pH 4 to 8. All aerobic growth experiments were performed in 96-well plates in a Cerillo Stratus plate reader placed in a shaking incubator set at 30 °C. The plate reader read OD600 once every hour. The shaking incubator was set to shake continuously at 200 rpm throughout each growth experiment.

### Anaerobic phenotyping conditions

For anaerobic growth, cultures grown in CEM, prepared as described above, were amended with 10 mM NaNO_3_. Anaerobic growth experiments included (1) the growth of *Castellaniella* isolates at various pHs (4–8) and (2) their growth with or without the FW104 COMM. For anaerobic growth experiments, 10 µL subsamples of overnight cultures were inoculated into 400 µL of CEM in individual wells of 100-well plates.  All anaerobic growth experiments were performed in a Bioscreen incubating plate reader (Thermo Labsystems) placed in an anaerobic chamber under a 78% N_2_/20% CO_2_/2% H_2_ headspace. The Bioscreen monitored growth by optical density measurements at 600 nm (OD600) once every hour at 30 °C. The Bioscreen was set to shake cultures continuously at low amplitude throughout each growth experiment. For experiments monitoring the growth of *Castellaniella* isolates with metal exposure like that observed in well FW104, a metal mix representative of FW104 contamination (FW104 COMM) was included at 1X concentration in specified cultures. The final 1X FW104 COMM contained 215 μM Al(SO­_4_)_2_ ∙ 12H_2_O, 80 µM C_4_H_6_O_6_U, 3000 µM MnCl_2_ ∙ 4H_2_O, 20 µM NiCl_2_ ∙ 6H_2_O, 2 µM CoCl_2_ ∙ 6H_2_O, 1 µM CuCl_2_ ∙ 2H_2_O, 10 µM Fe(SO_4_)(NH_4_)_2_(SO_4_) ∙ 6H_2_O, and 1 µM Cd(CH_2_CO_2_)_2_ ∙2H_2_O (Additional file 1: Table S1). The Al and U components were added from separate stock solutions, while the remaining metals were added from another 100X stock solution. This stock solution was stored in single-use aliquots at − 20 °C.

### Growth curve analysis

Analysis of growth curves was performed using the R (v4.2.3) package *gcplyr* (v1.5.2) as described in Blazanin [14] with specific parameters described below. Before the analysis of growth curve parameters, raw data were smoothed using the *moving-median* and *moving-average* smoothing algorithms. Additionally, OD600 values < 0.01 were excluded from further calculations to reduce the noise that arises at densities near 0. The per-capita growth rate (h^−1^) (i.e., the plain derivative divided by the population density) was calculated using the linear regression fitting functionality *window_width_n* of the *calc-deriv* algorithm with a window size of three data points.

### Nitrogen speciation and quantification

Nitrate reduction was assayed anaerobically using 10 mL of CEM containing 40 mM MES in Balch tubes with an 80% N_2_/20% CO_2_ headspace. Cultures were inoculated with 0.1–0.25 mL of overnight cultures and incubated as described above. Concentrations of nitrogen species were quantified as described previously [38]. Briefly, nitrate and nitrite were quantified by ion chromatography; ammonia was determined using the ammonia dichloroisocyanurate (DIC) assay; and nitrous oxide was quantified by direct headspace injection into a gas chromatograph.

### Curation and analysis of ORR amplicon sequence variant (ASV) data

Amplicon sequence variant data and associated geochemical data were retrieved from Goff et al. [38] and Ning et al. [75]. The Ning et al. [75] dataset includes ASVs generated from a groundwater sampling survey performed in July and November of 2012 alongside nitrate concentrations, pH, and multiple heavy metal concentrations. These samples were collected from wells drilled at the site. Briefly, the 16S V4 regions (253 bp in length) were amplified using the 515F/806R primer pair. Libraries were sequenced on an Illumina MiSeq platform. Amplicon sequence data from these prior studies were reprocessed to get ASVs for this study. ASVs were called using QIIME2 v. 2019.7 [16] and denoised and quality trimmed with the DADA2 plugin [20]. Taxonomy was called using the feature-classifier plugin with the Naive Bayes SILVA132 515F/806R classifier available on the QIIME2 website (https://docs.qiime2.org/2022.8/data-resources/). Mitochondria were removed from the data by removing any ASVs with “Mitochondria'' in their taxonomy. Chloroplasts were removed by removing any ASVs with “Chloroplast” in their taxonomy. We selected the subset of samples for our analysis. These samples were collected from areas immediately adjacent to the former S-3 pond (referred to as “Area 3”, “Area 1”, and “Area 5”) (Additional file 1: Fig. S2). Sequencing was performed on a 10.0 µm fraction representing particulate-matter-associated microorganisms and a 0.2 µm fraction representing planktonic microorganisms. The Goff et al. [38] dataset includes ASVs generated from a sediment core sampling survey performed in October of 2020 alongside porewater nitrate concentrations and sediment pH measurements [87]. These samples all originated from within Area 3, immediately adjacent to the former S-3 ponds.

To identify *Castellaniella* ASVs, we parsed the resulting ASV matrix for those with “*Castellaniella*” in their taxonomy. For the 2020 sediment survey, relative abundances of ASVs were averaged across the two replicate samples. For the 2012 groundwater survey [96], relative abundances of ASVs were averaged between the two size fractions to consider the overall distribution of the ASVs at the site. To perform correlational analyses with geochemical parameters, we only considered the 0.2 µm fraction from the 2012 groundwater survey. Pearson correlation coefficients and p-values were calculated with R (v4.2.3) in RStudio (v2022.02.1 Build 461) using the function *rcorr* in the *Hmisc* package (v5.0-1).

### Genome sequencing and curation of publicly available genomes

High-molecular-weight genomic DNA (gDNA) for strain MT123 was extracted using the Qiagen Genomic Tip 100/G kit according to the manufacturer’s protocol, except with the addition of 80 µL of Qiagen lytic enzyme solution during the first digestion step with lysozyme. This DNA was used as the input to both Nanopore sequencing and needle shearing followed by Illumina sequencing as described previously [37]. Data processing for Nanopore reads and Illumina reads was performed as described in Goff et al. [37]. Assembly was performed with the Nanopore and Illumina reads using Unicycler v0.4.8 as described in Goff et al. [37].

The gDNA of isolates FW104-7C03, FW104-12G02, FW104-16D08, FW104-7G2B, and GW247-6E4 were extracted using a Qiagen DNeasy kit according to the manufacturer's protocol for gram-negative bacteria. Illumina Libraries were constructed with ~ 250 ng of gDNA using an Illumina DNA Prep Tagmentation kit and Illumina DNA/RNA UD Indexes. Libraries were sequenced on an Illumina NovaSeq resulting in 2 × 150 paired end reads. The program Cutadapt v1.18 was used to remove adapter sequences [72], using the 3’ adapter sequence CTGTCTCTTATACACATCT. Sliding window quality filtering was performed with Trimmomatic v0.36 using parameters (-phred33 LEADING:3 TRAILING:3 SLIDINGWINDOW:5:20 MINLEN:50) [15]. All genomes were assembled de novo using SPAdes v3.15.3 with the following options (-k 21,33,55,77 –careful) [10]. Genome quality was validated with CheckM v1.0.18 using the *lineage_wf* pipeline with default parameters, maintaining only draft genomes with < 10% contamination and > 95% completeness [81]. The result is six high-quality draft genomes (< 60 contigs, N50 > 237,000). All the above programs were run using the US Department of Energy Knowledgebase (KBase) [5] using the KBase applications *kb_cutadapt* (v1.0.8), *kb_trimmomatic* (v1.2.13), *kb_SPAdes *(v1.3.3), and *kb_Msuite* (v1.4.0). All genomes were annotated using the “Annotate Multiple Microbial Assemblies with RASTtk” (v1.073) KBase application using default parameters [8, 18, 79].

Eight high-quality draft and completed *Castellaniella* sp. genomes were found through the NCBI and Joint Genome Institute (JGI) Integrated Microbial Genomes & Microbiomes (IMG/M) databases. This was the total number of high-quality genomes that were available as of September 2022. Genome quality was determined using the BV-BRC Comprehensive Genome Analysis Service [77]. Genomes annotated as “Good” quality by this tool were used for further analysis. Metadata including assembly length, isolation source, and sequencing information was also downloaded from IMG/M. The publicly available sequences were reannotated using the “Annotate Multiple Microbial Assemblies with RASTtk” (v1.073) application in KBase [5] using default parameters [8, 18, 79].

To identify ASVs matching the genomic 16S rRNA sequences, we retrieved 16S rRNA gene sequences from the *Castellaniella* genome annotations. These sequences were used to perform a BLASTn search [3] against the ASVs from the two prior surveys (described in the prior section).

### Phylogenetic analysis

To construct a phylogenetic tree that incorporates the ORR *Castellaniella* ASVs (Additional file 1: Table S2) identified in the ORR environmental samples (16S rRNA gene V4 region, 253 bp), we extracted complete 16S sequences from the sequenced *Castellaniella *genomes. Note that the three of the genomes retrieved from NCBI were metagenome-assembled genomes (MAGs) and did not contain usable 16S rRNA gene sequences for this analysis. Additionally, we retrieved the 16S sequences from NCBI for five reported *Castellaniella* species that are currently lacking genome sequences: *Castellaniella fermenti* CC-YTH191 (NR_159300.1), *Castellaniella hirudinis* E103 (NR_109550.1), *Castellaniella denitrificans* NKNTAU (NR_044802.1), *Castellaniella daejeonensis* MJ06 (NR_117260.1), and *Castellaniella ginsengisoli* DCY36 (NR_116482.1). First, an alignment was constructed with the ASVs and full-length 16S rRNA gene sequences using MUSCLE (v3.8.425) in Geneious Prime with default settings. Alignments were trimmed to the 253 bp partial sequence. This trimmed alignment was used to generate a maximum likelihood tree with PhyML (v3.3.20180214) [41] using an HYK85 nucleotide substitution model in Geneious Prime (v2022.0.2). Branch support was determined with 1000 bootstraps. Otherwise, default parameters were used. To determine the root of this tree, a second tree was constructed using the same method but including six outgroup 16S rRNA gene sequences from *Advenella mimigardefordensis* DPN7, *Alcaligenes faecalis* subsp. faecalis NBRC 13111, *Pusilimonas noertemannii* BS8, *Bordetella pertussis* Tohoma I, *Achromobacter oxylosoxidans* NBRC 15126, and *Bordetella petrii* DSM 12804. The original tree was re-rooted based on the results of this second tree containing the outgroup sequences. All phylogenetic trees in this study were visualized using iToL [67].

We also constructed a full-length (~ 1550 bp) 16S rRNA gene tree using all publicly available *Castellaniella* full-length 16S sequences as well as full-length 16S sequences from *Advenella mimigardefordensis* DPN7, *Alcaligenes faecalis* subsp. faecalis NBRC 13111, *Pusilimonas noertemannii* BS8, *Bordetella pertussis* Tohoma I, *Achromobacter oxylosoxidans* NBRC 15126, and *Bordetella petrii* DSM 12804. Sequences were aligned, as described above with MUSCLE. This alignment was used to generate a maximum likelihood tree, as described above, with PhyML.

A multi-locus tree was constructed in KBase with the 14 *Castellaniella* genomes using the Insert Set of Genomes Into SpeciesTree application (v2.2.0) [86]. The marker sequences for building the concatenated alignment are given in Additional file 1: Table S3. Average Nucleotide Identity (ANI) was also calculated for these genomes using the Compute ANI with the FastANI [53] application in KBase.

### Pangenome analysis

A pangenome comparing eight publicly available *Castellaniella* sp. genomes with six additional genomes presented herein (for a total of 14 genomes) was computed in KBase using the “Compute Pangenome” (v0.0.7) application with default methods [47]. This program uses protein 8-mers to cluster ortholog families and has been applied to recently described pangenome analyses [46, 51, 107]. Genes were categorized as part of the core (present in 100% of genomes), shell (present in 16–99% of genomes), or cloud (present in less than 1–15% of genomes).

#### Functional annotations

The gene families from each genome were functionally characterized using the COG functional categories with eggNOG-mapper (v2.1.9) under default parameters [50]. The genomes were analyzed for heavy metal homeostasis genes (HMHGs) using Geneious Prime (v2022.0.2) to perform a BLASTp search against the BacMet Antibacterial Biocide and Metal Resistance Genes Predicted Database (v2) [80]. Curation of the results followed: (1) results with an e-value > 1E−10 were discarded; (2) results with a query coverage < 70% were discarded; (3) results with a pairwise identity < 25% were discarded; and (4) results with 25% < pairwise identity < 80% were manually assessed for likelihood of a positive hit based on sequence length, similarity of the protein annotation, and a BLASTp [3] search against the UniProtKB/SwissProt database [25]. A manual review of annotations identified genome features associated with the denitrification process. Denitrifying genes were identified manually in accordance with previous reports [64, 103].

#### Chromosomally integrated element analysis

The web-based software, IslandViewer 4 [12] was used to broadly identify genomic islands (integrating two different methods: SIGI-HMM [106] and IslandPath-DIMOB [48]). Further classification of IslandViewer4 predictions was conducted using PHage Search Tool Enhanced Release (PHASTER) [6, 108] and ICEFinder [69] to identify prophage regions and integrative and conjugative elements, respectively.

#### Castellaniella global geography

*Castellaniella* 16S rRNA gene sequences were retrieved from the SILVA database (v.138.1) in August 2023. Associated metadata were used to determine the environmental classification of and anthropogenic impact on the environmental sites of origin of these sequences. Our environmental classification scheme was based on the JGI GOLD ecosystem ontology [74]. For mapping, we extracted coordinates and/or location information from the metadata.

#### Other data visualization

Heatmaps were visualized using the *pheatmap* (v1.0.12) package [61] in R (v4.2.3) in RStudio (v2022.02.1 Build 461). Correlelograms were visualized using the *corrplot* (v0.92) R package. Other plots were generated using the *ggplot2* (v3.4.2) R package.

## Results and discussion

### Castellaniella diversity, distribution, and history in the ORR subsurface

**Fig. 1:**
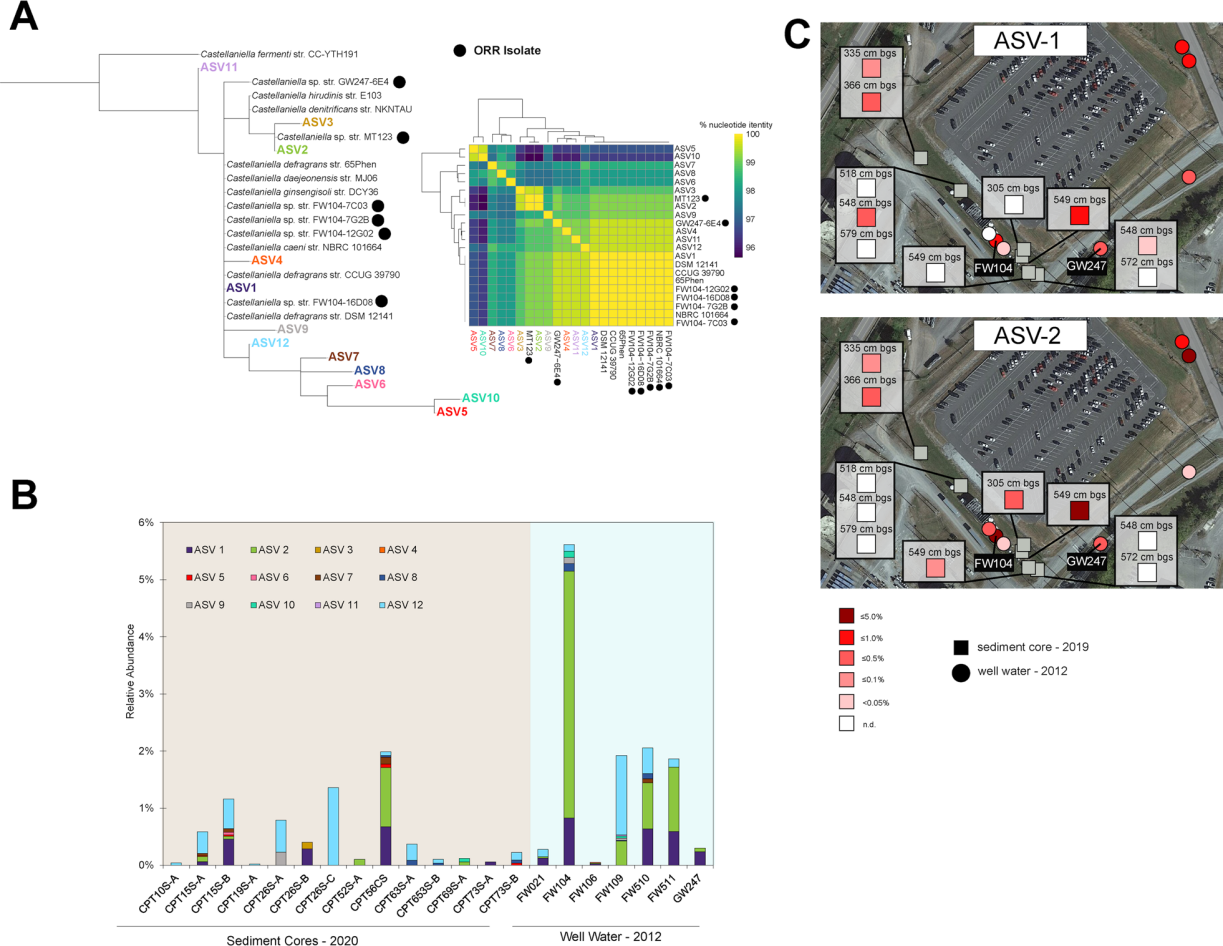
Ecology of ORR *Castellaniella* populations. **A** Maximum-likelihood tree of *Castellaniella* 16S rRNA gene V4 regions, incorporating the ORR ASVs (left) and a matrix showing the percentage nucleotide identity for the same set of sequences (right). ORR isolates are indicated with a black circle. The associated alignment is shown in Additional file 1: Fig. S2. **B** Relative abundances of *Castellaniella* ASVs around the former S-3 ponds. Only samples with detectable *Castellaniella* ASVs are shown. **C** Spatial distributions in the ORR subsurface of two dominant ASVs with cultured representatives. Map data provided by Google Maps ©2023 Airbus, Maxar Technologies, U.S. Geological Surveym USDA/FPAC/GEO

Using 16S rRNA (V4 region) gene amplicon sequence variants (ASVs) obtained in prior community analyses [38, 75, 96], we examined the geographic range of *Castellaniella* in the region of the ORR subsurface surrounding the former S-3 ponds (Additional file 1: Fig. S1). We detected 12 *Castellaniella* ASVs across these samples (Additional file 1: Table S2, Fig. 1A, B, Additional file 1: Fig. S2). Of these 12 ASVs, three were found to exceed a relative abundance of 0.5% in one or more samples: ASV1, ASV2, and ASV12. These three ASVs were present to varying degrees in the earlier community surveys, with ASV2 reaching the highest average relative abundance at 4.3% of the well FW104 groundwater ASVs.

To link genotypic and phenotypic analyses with field observations, we isolated six *Castellaniella* strains from the contaminated ORR groundwater: FW104-16D08, FW104-12G02, FW104-7G2B, FW104-7C03, GW247-6E4, and MT123 (Additional file 1: Table S4). Strain GW247-6E4 originated from the contaminated well GW247 while the remaining strains all originated from the contaminated well FW104. FW104 is the location of the *Castellaniella* bloom described above that was observed in the 2012 survey [96]. The strain MT123 V4 region matches ASV2 at 100% nucleotide identity. The V4 regions of strains FW104-16D08, FW104-12G02, FW104-7G2B, and FW104-7C03 match with 100% nucleotide identity to ASV1. ASV1 is also a 100% match to the 16S rRNA gene V4 regions of *C. ginsengisoli* (ginseng field soil) [59]*, C. daejeonensis* (oil-contaminated soil) [66], and *C. defragrans* (pristine and oil-contaminated soils) [58, 84]*.* However, the ORR ASV1 population is likely to represent a distinct ecotype from these close relatives, given the differences in their environments of origin [24]. The GW247-6E4 V4 region did not match at 100% identity to any of the 12 *Castellaniella* ASVs (Fig. 1A, Additional file 1: Fig. S2). While ASV12 is generally found at higher abundance than ASV1 (Fig. 1A), unfortunately we were unable to recover a cultured representative of this population.

We next mapped the abundance patterns in the ORR subsurface of the two ASVs with representative isolates (ASV1 and ASV2) (Fig. 1C). As described above, there was a focal hotspot of both ASV1 and ASV2 in the FW104 groundwater in a 2012 groundwater survey [96]. ASV1 and ASV2 were also observed at higher abundance in a nearby sediment core during a later 2020 survey [38]. These data suggest that these two *Castellaniella* clades stably coexist in the ORR subsurface.

Biostimulation efforts conducted around the former S-3 ponds in the early 2000s induced *Castellaniella* blooms and enhanced denitrification rates [98, 99]. Spain et al. [98, 99] reported on a 16S rRNA gene clone library from an ORR sediment core collected after biostimulation with ethanol and bicarbonate. In this core, two *Castellaniella* clones dominated: “Operational Taxonomic Unit (OTU) 34” (70% relative abundance) and “OTU35” (11% relative abundance). Comparisons to the V4 region of these clones revealed that ASV2 has 100% sequence identity to the V4 region of OTU34, except for a single nucleotide ambiguity (Additional file 1: Fig. S3). Additionally, the ASV2-representative MT123 16S rRNA gene sequence has 100% nucleotide identity to the full-length OTU34 sequence except for the single nucleotide pair ambiguity (Additional file 1: Fig. S4).

MT123 is a representative of a *Castellaniella* lineage that has persisted for at least two decades across multiple locations at this site, undergoing periodic blooms in response to the modulation of its local environmental chemistry. Such extreme persistence is unusual among microbial populations inhabiting dynamic environments like the ORR subsurface. Sporadic biostimulation events [1, 26, 55, 98, 99], other site remediation efforts [52], and rainfall events impacting groundwater flow and contaminant transport [82] have created an environment where major perturbations to the ORR subsurface microbial community are common. Prior studies have found that soil microbial communities are typically sensitive to disturbances [2]. Community stability is dependent upon the persistence of microbial populations, such as the different *Castellaniella* ASVs discussed here [94]. However, our results suggest that the ASV2 population is highly persistent despite occupying this frequently disturbed environment. Rapid evolution through the acquisition of mobile genetic elements via horizontal gene transfer can be one mechanism through which populations ensure persistence [7, 9, 54]. Population-level persistence is also governed by the survival of individual cells, which is controlled by factors such as dormancy, physiological plasticity, and the inventory of available stress response mechanisms [94]. Thus, we predicted that genomic features, specifically horizontally acquired genetic material, and laboratory phenotypes of these representative strains could be used to inform the controls on the distribution of these two dominant *Castellaniella* ASVs at the site and the persistence of the ASV2 population, in particular.

### A refined *Castellaniella* phylogeny

**Fig. 2:**
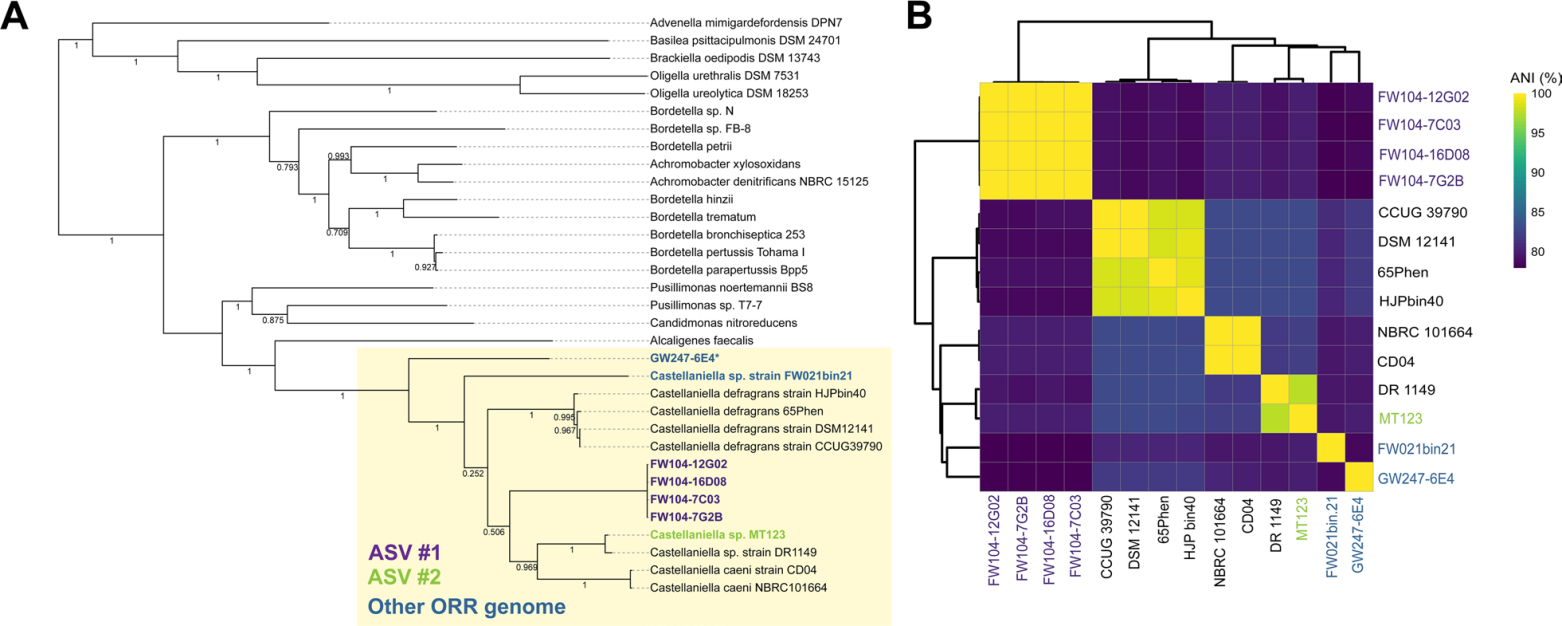
*Castellaniella* phylogeny. **A** Multi-locus phylogenomic tree of *Castellaniella* and close relatives using a concatenated alignment of 49 single-copy marker genes (Additional file 1: Table S3). Bootstrap values are shown on the tree. **B** Average nucleotide identity (ANI) matrix of *Castellaniella* genomes clustered using a Euclidian distance metric. ORR genomes are highlighted by different colored text (purple for ASV1-matching strains, green for ASV2-matching strains, and blue for other ORR genomes)

Our initial analysis using the 16S V4 region (Fig. 1A) had poor resolution of multiple previously described *Castellaniella* species and ASV1, suggesting that the 16S V4 region may significantly underestimate *Castellaniella* diversity. This is in line with other recent studies that have highlighted the low discriminatory power of this region [43, 56]. Thus, we sought to use sequenced *Castellaniella* genomes to refine the taxonomic placement of our ORR isolates. In addition to our six sequenced ORR *Castellaniella* genomes, we included eight high-quality *Castellaniella* genomes (Additional file 1: Table S4, Fig. S5). Six of these genomes originated from sludges or bioreactors of wastewater treatment plants where physiological stressors such as high concentrations of antibiotics and nitrates are commonly found [29, 63]. One of these genomes, FW021bin21, is a MAG originating from ORR well FW021, located adjacent to the former S-3 ponds (see Area 1, Additional file 1: Figure S1). ANI analysis for all *Castellaniella* genome pairs revealed six distinct clades with ANI values greater than 95% within the clades. We consider each of these clades to represent different *Castellaniella* species [39]. This phylogeny is further supported by a multilocus species tree with all fourteen genomes (Fig. 2A, B). While we had hypothesized that the genomes derived from ORR would cluster with each other given their shared environmental origin, this was not the case. Based on the multilocus species tree and matrix of ANI values, only one clade contained multiple ORR genomes: ASV1 representatives FW104-16D08, FW104-12G02, FW104-7G2B, and FW104-7C03, all originating from FW104. The ASV2-representative genome, MT123, forms a clade with DR1149, originating from an anaerobic digester. The two other ORR genomes, FW021bin21 and GW247-6E4, formed singleton clades (Fig. 2B). Thus, despite originating in close physical proximity, the ORR *Castellaniella* genomes form four distinct clades, including three that likely represent novel *Castellaniella* species.

Several validly described *Castellaniella* species—*C. denitrificans* [58]*, C. ginsengisoli* [59], *C. daejeonensis* [66], *C. hirudinis *[35]*,* and *C. fermenti* [68]*—*have no reported genome sequences and thus were excluded from this analysis. Other studies have reported various full-length 16S rRNA gene trees to define the phylogeny of the genus [35, 58, 59, 66, 68]. In line with these studies, we generated a full-length 16S gene tree to examine the relatedness of our strains to those without genomes (Additional file 1: Fig. S6). The novel ORR genomes form distinct clades from all other reported *Castellaniella* species. However, the usage of 16S rRNA gene percentage identity for defining species cutoffs is controversial, and specific cutoff values are likely genus-dependent [13, 92]. For example, if the conventional 97% metric is used, only *C fermenti* would be considered its own species. Additionally, as single gene trees cannot be used as species trees [34, 57], efforts should be taken in the future to ensure completed genome sequencing of this *Castellaniella* diversity.

### *Castellaniella* pangenome structure

**Fig. 3:**
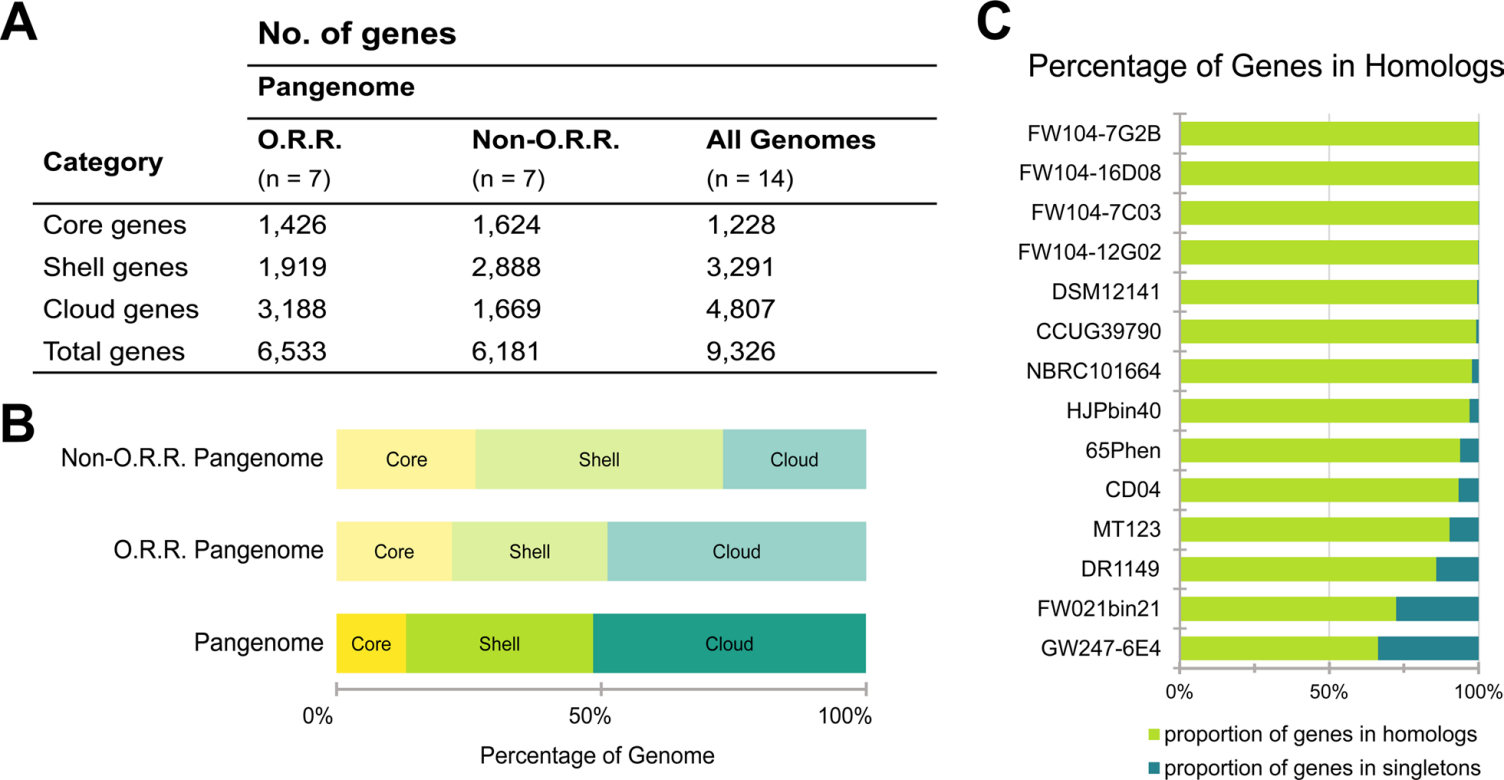
*Castellaniella* Pangenome. **A** The number of orthologs in each of the pangenome categories (core, shell, and cloud). The table columns represent the genomes included in the pangenome analysis. ORR signifies the pangenome was computed only using genomes derived from Oak Ridge Reservation whereas non-ORR included any publicly available *Castellaniella* genomes that did not originate from this site. **B** The proportion of orthologs in each category with respect to the genomes included in the pangenome analysis. The core (yellow) category is made up of orthologs that are present in all genomes. The shell (green) category includes orthologs found in 16 ≤ x < 95% of genomes. The cloud (turquoise) category represents orthologs that are found in < 16% of the genomes included in the analysis. **C** The distribution of homologs and singleton genes in the 14 *Castellaniella* genomes

A *Castellaniella* pangenome was computed using the 14 genomes to contextualize our ORR isolates within the genetic diversity of this genus. The resulting pangenome contained 9,326 unique orthologous gene clusters (orthologs) that were categorized based on the percentage of genomes containing the given ortholog. The *Castellaniella* pangenome (n = 14) contained 1228 core gene clusters (13% of the pangenome), 3291 shell gene clusters (35%), and 4807 cloud gene clusters (51%) (Fig. 3A). Consistent with the greater taxonomic diversity of the ORR isolates, the ORR pangenome has a larger cloud genome (49% of the pangenome) than the non-ORR pangenome (27%) (Fig. 3B, C). However, the genetic diversity of the *Castellaniella* genus is extremely undersampled (as evidenced, in part, by our discovery of what are likely two novel species in a relatively small geographic range), thus it is challenging to make extensive comparisons between these two datasets.

Cloud genes are frequently associated with mobile genetic elements (MGEs). MGEs were found in all fourteen genomes with varying sizes and gene content, much of which is part of the *Castellaniella* cloud and shell genomes (Additional file 2: Table S5). The acquisition of these elements by horizontal gene transfer paired with their integration into the host's genome is likely a factor contributing to the adaptation of these microorganisms to the unique environments at the ORR. There was no significant difference (*Student’s* *t* test*, p* < 0.05) in the total predicted MGE content between the ORR and non-ORR genomes, suggesting a similar propensity across the genus to horizontally acquire novel genetic material (Table 1). The MGEs most common across all the *Castellaniella* genomes were genomic islands (GIs). Since the definition for GIs is broad, many elements that do not fit the criteria for other MGE types (e.g., integrative and conjugative elements, phages, insertion sequences) end up being classified as GIs [65]. The ASV1-representative genomes had greater predicted numbers of MGEs than the ASV2-representative genome MT123 (Table 1). However, the proportions of the different types of MGEs varied from individual genome to genome.

**Table 1:** *Castellaniella* chromosomally integrated elements

Genome	Location of origin	Total predictions	Proportion GI (%)	Proportion ICE (%)	Proportion prophage (%)	Proportion IS (%)
HJPbin40	Non-ORR	15	87	0	0	0
65Phen	Non-ORR	22	32	9	18	5
DSM12141	Non-ORR	21	57	19	5	0
CCUG39790	Non-ORR	22	41	18	5	0
DR1149	Non-ORR	24	33	4	25	0
CD04	Non-ORR	26	46	8	15	0
NBRC101664	Non-ORR	23	43	13	4	0
	**AVG**	**21.86**	**49**	**10**	**11**	**1**
	**SD**	**3.44**	**19**	**7**	**9**	**2**
GW247-6E4	ORR	20	40	10	0	0
FW021bin21	ORR	20	25	15	20	5
FW104-12G02	ORR	27	26	41	7	0
FW104-16D08	ORR	27	41	11	11	7
FW104-7C03	ORR	27	41	0	11	4
FW104-7G2B	ORR	26	46	4	8	12
MT123	ORR	19	26	16	5	0
	**AVG**	**23.71**	**35**	**14**	**9**	**4**
	**SD**	**3.82**	**9**	**13**	**6**	**4**

The total number of predictions made for each genome by IslandViewer4 as well as the percentage of these predictions further classified by additional annotation tools as being genomic islands (GIs), integrative and conjugative elements (ICEs), prophages or insertion sequences (ISs)

### Key functional traits for persistence in the ORR subsurface

Focusing on the ORR strains representing *Castellaniella* ASV1 (FW104-16D08, FW104-12G02, FW104-7G2B, FW104-7C03) and ASV2 (MT123), we examined key functional traits that may facilitate their survival and distribution at the site. We sought to determine which of these traits were associated with the identified MGEs and to what extent those elements may shape the fitness of the *Castellaniella* populations at this site.

#### Acid tolerance

Bacterial populations in the ORR subsurface experience a wide range of pH, from acidic to neutral. In other studies, pH is a significant variable in determining the geographic range of microorganisms [31, 89, 96]. Thus, we sought to examine the pH range permissive to the growth of our ASV1- and ASV2-matching ORR isolates to understand how pH may control their distribution in the ORR subsurface. In locations where the relative abundance of ASV1 and ASV2 is 0.5% or greater, both have similar environmentally defined pH ranges: ASV1 (3.9–7.4) and ASV2 (4.0–7.0). This is consistent with prior studies which have found that pH preference in situ is deeply conserved in prokaryotic lineages [73]. The maximal relative abundance of both ASVs occurs in FW104 which has an average pH of 5.6. This observation is consistent with the pH optima for the ASV1-matching strains FW104-12G02 (pH 5.5), FW104-7C03 (pH 5.5), and FW104-7G2B (pH 5.5–7.0) (Additional file 1: Table S6). However, ASV1-matching strain FW104-16D08 and ASV2-matching strain MT123 had higher pH optima, around pH 6–8 and pH 7–8, respectively. Nonetheless, both strains still grew robustly at pH 5.5, with MT123 having its highest carrying capacity at pH 5.5 despite the slower growth rate, exceeding that of the ASV1 representatives at this pH. Thus, we propose that, at least within the genus *Castellaniella*, there are strain-level differences in pH optima even though, as the genus, the pH range permissive to growth is well-conserved.

Only FW104-12G02, FW104-7G2B, and FW104-7C03 had significant growth at pH 5, a common condition across the contaminated subsurface, and none of the strains had significant growth at pH < 5. In the field, the populations represented by ASV1 and ASV2 may persist in a dormant state < pH 5 and undergo periodic blooms as pH increases, as was observed in the Spain et al. [98, 99] study when the subsurface pH was raised with bicarbonate. Additionally, our usage of batch cultures for determining pH tolerance may result in an underestimation of the pH range permissive towards growth [38]. Indeed, the permissive pH range for MT123 growth in laboratory batch culture extends down to 5.0 when grown aerobically without nitrate (Additional file 1: Fig. S7), eliminating the accumulation of the protonophore nitrous acid [95].

We next examined the acid-tolerance systems of these key ORR isolates in the context of the *Castellaniella* pangenome (Additional file 1: Fig. S8). In general, *Castellaniella* genomes have a small repertoire of known acid tolerance genes, in line with the observation that our isolates only tolerate weakly acidic conditions in the laboratory. The *Castellaniella* core genome includes the following genes known to be associated with low pH tolerance (but not exclusively involved in low pH tolerance): (1) RecA for DNA repair [101], (2) DnaK for protein re-folding [105], (3) the Pst phosphate transporter [93], and (4) genes involved in the arginine-dependent low pH tolerance system [93]. MT123 encodes an additional aspartate-based [49] low pH tolerance system while FW104-12G02, FW104-7C03, FW104-7G2B, and FW104-16D08 encode a genome island-associated glycerol-3-phosphate transporter [93]. While the urease system is a commonly described acid tolerance mechanism [71], neither the ASV1 nor ASV2-representative genomes encode these genes. This system was not observed in any of the analyzed *Castellaniella* genomes except the ORR MAG FW021bin21. This MAG originates from well FW021 (see Area 1, Additional file 1: Fig. S1), which has among the lowest recorded pH values (3.4) at the site [102]. However, given that there are no genes exclusively implicated in acid tolerance reported in the literature [71], it is challenging to draw a straightforward connection between gene number and/or specific gene content and an organism’s permissive pH range or optima.

#### Denitrification activity

**Fig. 4:**
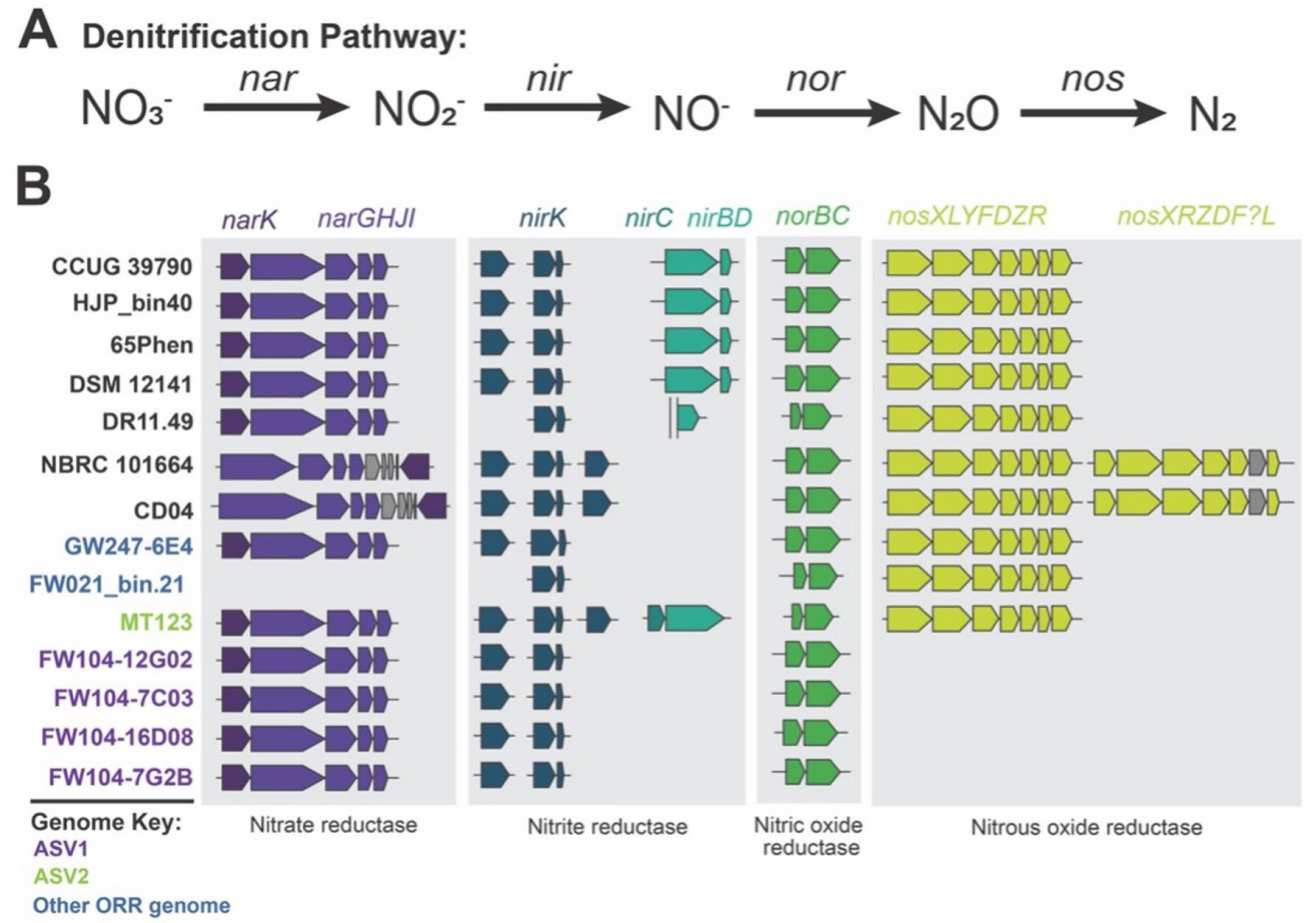
Denitrification Pathway. **A** Schematic representation of the denitrification pathway and the genes associated with each step. **B** The distribution of denitrification genes in the fourteen *Castellaniella* genomes. Non-ORR genomes are listed in black and ORR genomes are colored according to their matching ASV where purple represents ASV1 matching genomes, green represents the ASV2 matching genome and blue represents genomes which do not match either ASV. Genes shown include those encoding the nitrate/nitrite transporter (*narK*), the respiratory nitrate reductase and associated assembly proteins (*narGHJI*), the copper-containing respiratory nitrite reductase (*nirK*), the nitrite transporter (*nirC*), the NAD(P)H nitrite reductase (*nirBD*), the cytochrome c-dependent nitric oxide reductase (*norBC*), and the nitrous oxide reductase and associated assembly proteins (*nosXLYFDZR*)

Classically, *Castellaniella* are characterized as complete denitrifiers, reducing nitrate to dinitrogen gas [58]. Indeed, we observed that the ASV2-representative MT123 reduces nitrate to dinitrogen gas with no accumulation of nitrous oxide. MT123 NosZ was active in both the pH 7 and pH 5.5 cultures (Additional file 1: Table S7). Based on bioinformatic analyses, the MT123 NosZ is a Clade II type [42]. Interestingly, in many Clade II denitrifiers, NosZ activity is eliminated below pH 6–6.5 [21, 76]. In our analysis, we also identified several variants on the *Castellaniella* denitrification pathway. For example, the ORR MAG, FW021bin21, lacks the nitrate reductase operon (*narGHJI*) as well as the nitrate/nitrite transporter gene (*narK*) (Fig. 4A, B). Interestingly, the nitrous oxide reductase operon (*nosXLYFDZR*) for this MAG were found within a region predicted to be a genomic island. Six of the *Castellaniella* genomes have the NAD(P)H-dependent nitrite reductase genes (*nirBD*) (Fig. 4B). Out of those six genomes, only ORR genome MT123 contained the nitrite transporter gene *nirC*. Two of the non-ORR genomes, NBRC101664 and CD04, have a second copy of the nitrous oxide reductase operon (*nosXLYFDZR*) (Fig. 4B).

Perhaps the most extreme instances of denitrification pathway varianst in our analysis are the four ASV1-matching strains that lack the *nos* operon (Fig. 4B). The *nos* operon locus is highly conserved among *Castellaniella* and is adjacent to an arginine tRNA gene. Our analysis of this highly conserved region of the genome in ASV1-matching strains revealed that copper resistance genes had been inserted at this location as part of a larger genomic island. tRNA genes are common insertion sites for mobile genetic elements in microbial chromosomes [17, 104]. The acquisition of these copper resistance genes may contribute to the survival of these ASV1-matching strains within the subsurface at ORR. However, the absence of the *nos* operon in these four ASV1-matching strains suggests the final step in their dentification pathways is the production of the greenhouse gas nitrous oxide. Using strain FW104-7G2B as a representative, we confirmed the reduction of nitrate to nitrous oxide without further reduction to dinitrogen gas (Additional file 1: Table S7). We propose that, despite the acquisition of copper resistance genes, this inability to perform nitrous oxide reduction contributes to the apparent lower fitness of ASV1 at the site relative to ASV2 (Fig. 1B, C). ASV1 strains are predicted to have lower molar growth yields from partial denitrification compared to complete denitrifiers like MT123 [60]. Additionally, nitrous oxide is cytotoxic. Its accumulation can inactivate vitamin B12-dependent enzymes involved in methionine and DNA biosynthesis [100]. ASV1 strains may be heavily dependent on nitrous oxide-consuming neighbors to relieve this toxicity.

#### Heavy metal homeostasis

There is emerging evidence to suggest that metals in combination interact synergistically or in an antagonistic manner within bacterial systems [36, 85]. As the ORR subsurface is contaminated by complex and heterogenous mixtures of metals, we tested microbial resistance to the metal composition present in the FW104 well **(**Additional file 1: Table S1), the site of origin for our ASV1 and ASV2 representative strains. The ASV2 representative MT123 experienced only a minor growth defect with the metal mixture, specifically, an extended lag phase. In contrast, the growth of the ASV1 representatives FW104-16D08, FW104-12G02, FW104-7G2B, FW104-7C03 was significantly inhibited by the FW104 metal mixture (Additional file 1: Fig. S9). These findings add context to the observation that ASV2 is at a greater abundance in the FW104 groundwater (and across all samples) relative to ASV1 (Fig. 1B, C). Metal tolerance may be a significant distinguishing factor between co-existing *Castellaniella* populations in the ORR subsurface.

**Fig. 5:**
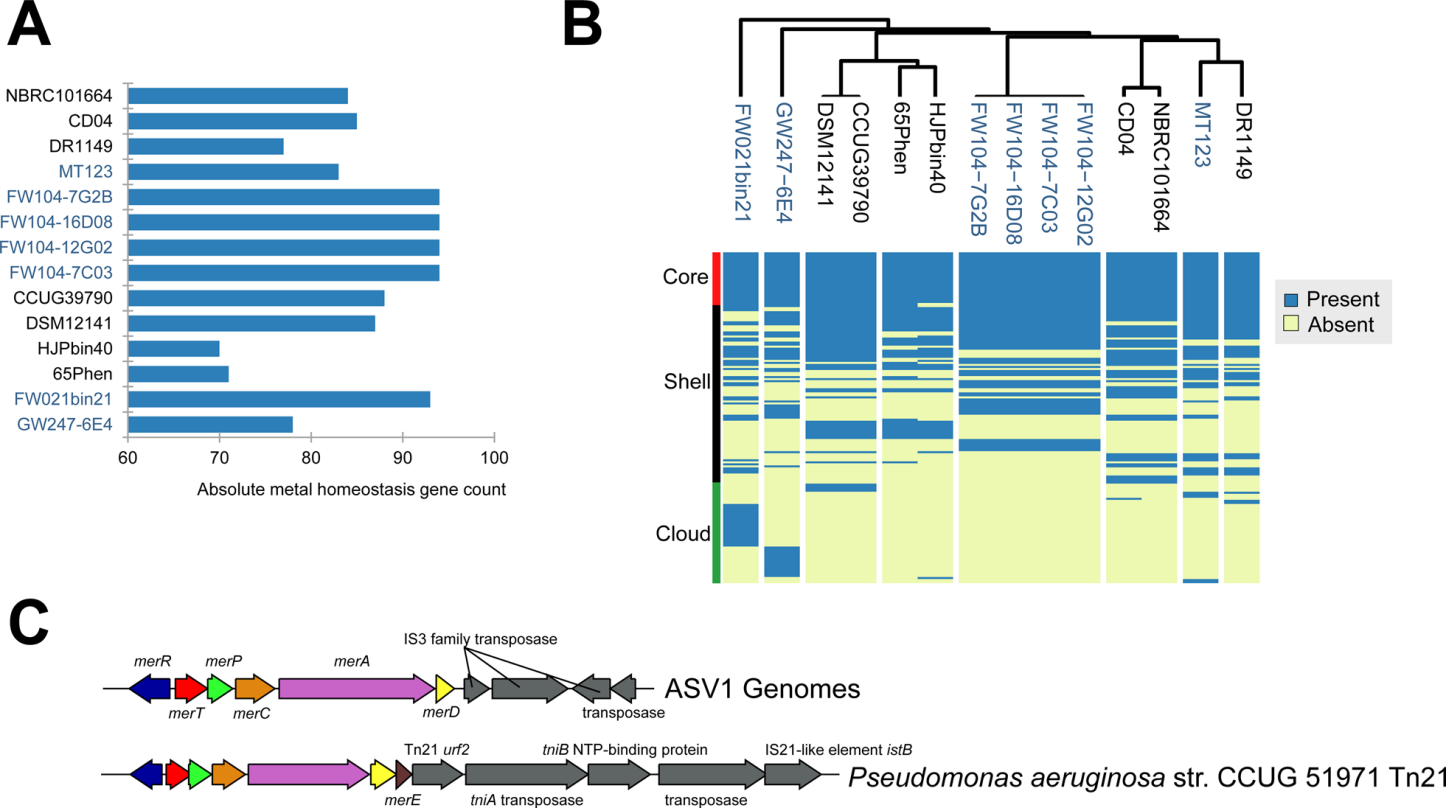
Metal homeostasis genes. **A** Counts of heavy metal homeostasis genes (HMHG) identified in *Castellaniella* genomes. **B** Presence/absence matrix of individual HMHGs in *Castellaniella* genomes. Whether a particular gene belongs to the core, shell, or cloud genome is indicated. Clustering was performed using a Euclidian distance metric **C** Mercury transposon observed in the ASV1-representative genomes compared to a canonical *Tn*21 mercury transposon in *P. aeruginosa*

We examined heavy metal homeostasis gene (HMHG) content in the *Castellaniella* pangenome: encompassing genes involved in the import, export, intracellular trafficking, and transformation of metals [22]. ORR genomes encode an average of 90 HMHGs compared to 80 in non-ORR genomes (Student’s two-sided *t* test p < 0.05) (Fig. 5A). However, this difference is likely driven by the four closely related ASV1-representative genomes which each have a large inventory of HMHGs. Although the ORR MAG FW021bin21 also has a large repertoire of HMRGs. Among the ORR genomes, the strain GW247-6E4 genome had the lowest HMHG count. Notably, this strain is not reflected in any of the ORR *Castellaniella* ASVs, suggestive of its low relative abundance at the site. However, when comparing the ASV1 and ASV2-representative strains, we found that the total number of HMHGs did not correlate with their degree of metal mixture tolerance (Additional file 1: Fig. S9). Instead, we propose that the presence or absence of specific HMHGs drives the differences in metal tolerance between these two ORR populations. The MT123 genome encodes 17 HMHGs not found in any of the ASV1-representative genomes (Additional file 1: Table S8). These include two P-type metal ion-pumping ATPases and several copper and mercury resistance genes.

Cloud and shell HMHGs are frequently associated with mobile genetic elements [91] (Fig. 5B). Within the ORR genomes, 0–40% (median = 16%) of the identified HMHGs are located on mobile genetic elements. These are similar values to what was previously observed for *Rhodanobacter* from the ORR subsurface [83]. For example, the copper resistance genes inserted at the location of the *nos* operon of the ASV1-representative genomes are part of a larger predicted genome island (GI). We also identified a mercury resistance transposon containing the *merRTPCAD* operon in the ASV1-representative genomes (Fig. 5C). This *mer* operon resembles the well-characterized gram-negative Tn*21,* Tn*501,* and pKLH2 mercuric ion resistance operons [78]. Tn*21* and Tn*501* were both shown to be highly abundant in the mercury-contaminated New Hope Pond site near the former S-3 ponds [11]. Mercury is also part of the S-3 contaminant profile [62, 90]. We also found that most of the ASV2-specific HMHGs genes described above (Fig S9) are localized to a single GI. Given the greater resistance of MT123 to the FW104 metal mixture compared to the ASV1-representative strains, acquisition of this GI may have increased the ASV2 population fitness within this niche relative to the ASV1 population (Additional file 1: Table S5). Overall, the accessory HMHG profiles are consistent with the phylogenomic tree shown in Fig. 2A, with more closely related genomes having more similar shell and cloud HMHG content, suggesting that these traits are conserved on a very fine-grained level (Fig. 5B). This is consistent with recent findings that host phylogeny significantly controls the acquisition of novel functional traits like resistance genes [32].

### Global significance

**Fig. 6:**
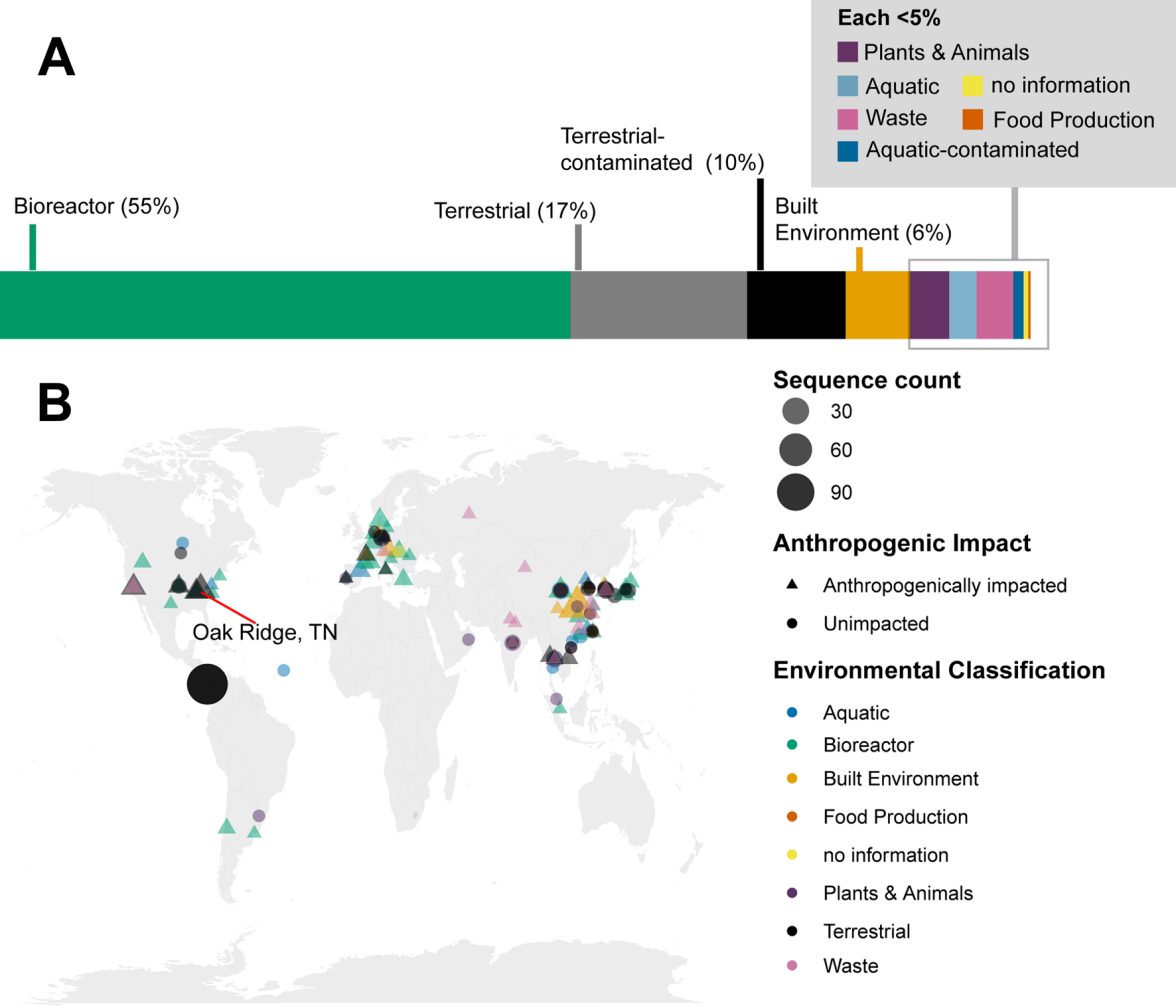
Global distribution of *Castellaniella* 16S rRNA gene sequences. *Castellaniella* 16S rRNA gene sequences were retrieved from the SILVA database (v.138.1). **A** The bar graph shows the percentages of 16S rRNA gene sequences (n = 898 total) associated with each environment classification. **B** The map shows the geographic distribution of the 16S rRNA gene sequences. Colors indicate the environmental classification. Shapes indicated sites that are anthropogenically impacted (triangle) or unimpacted (circle). Shapes are scaled to reflect the count of sequences originating from a single location

The majority of sequenced *Castellaniella* genomes analyzed in this study originate from anthropogenically impacted sites like the ORR subsurface and anaerobic digesters. We downloaded an additional 898 *Castellaniella* 16S rRNA gene sequences from the SILVA database (Fig. 6A, Additional file 1: Table S8). Of these, 680 (76%) originate from globally distributed anthropogenically impacted sites (Fig. 6A, B) that we broadly categorized as “Terrestrial-Contaminated”, “Aquatic-Contaminated”, “Solid Waste”, “Built Environment”, and “Wastewater”. We propose that this genus is specialized for growth in sites, which are typically impacted by multiple stressors simultaneously (i.e., heavy metals, organics, nitrogen pollution, and/or low pH). This is likely due, in part, to the ability of members of this genus to acquire novel genes via HGT, which may allow for rapid adaptation to their local niche. For example, in addition to HMRGs, many of the genomic islands observed in the *Castellaniella* genomes (both ORR and non-ORR) carry genes involved in acid tolerance, aromatic carbon degradation, toxin/antitoxin defense systems, and biofilm formation. However, as we show here, any fitness improvements from the acquisition of these mobile genetic elements are likely controlled by (1) the insertion location of the element and (2) the specific genes acquired.

## Conclusions

Blooms of the denitrifier genus *Castellaniella* have occurred in the recent decades in the contaminated subsurface at the ORR. Here we demonstrate that one of these *Castellaniella* population has persisted for decades in the ORR subsurface. Analysis of the genomes of ORR *Castellaniella* strains suggest that these lineages have a high propensity to horizontally acquire and integrate novel genetic material into their genomes. Investigation of the representative functions for genes encoded within these chromosomally integrated regions in the ORR genomes revealed acid tolerance, denitrification, and heavy metal homeostasis genes, which may increase the fitness of these populations at this site. These data demonstrate the importance of horizontal gene transfer in the diversification and adaptation of microorganisms in this multi-stressor environment. The discussion of niche-specific adaptations raises the question of whether contaminants found in the subsurface prompted these differentiation events through increased rates of horizontal gene transfer [23] or whether the isolates already harbored the functionalities necessary to allow for their survival in such an environment [45]. The answer likely falls somewhere between those two scenarios. On-going mutant fitness and omics-based analyses of these ORR *Castellaniella* isolates seek to further characterize the genomic controls of *Castellaniella* persistence at ORR.

### Supplementary Information

**Additional file 1**. Supporting Information.

**Additional file 2**. Supplementary Table S5.

## Data Availability

Assembled genomes and annotations can be found in the KBase narrative: 10.25982/135835.33/2203552. The narrative also contains the output files for the pangenome analysis. All genomes and Illumina raw reads are available on NCBI as BioProject PRJNA1100609.
